# Carcinoma In Situ Is Significantly Underdetected by Prenephroureterectomy Ureteroscopy in the Management of Upper Tract Urothelial Cancers

**DOI:** 10.1155/2015/547586

**Published:** 2015-01-15

**Authors:** Angela Gillan, Ismail El-Mokadem, Bhavan Rai, Stephen Lang, Jason Alcorn, Altaf Shams ud din, Ranan Dasgupta, Chandra Shekhar Biyani, Ghulam Nabi

**Affiliations:** ^1^Academic Section of Urology, Division of Imaging and Technology Medical Research Institute, University of Dundee, Dundee DD1 9SY, UK; ^2^Department of Pathology, Ninewells Hospital, NHS Tayside Health Board, Dundee DD1 9SY, UK; ^3^Department of Urology, Pinderfield General Hospital and Mid Yorkshire NHS Trust, Wakefield, UK; ^4^Department of Urology, Charing Cross Hospital, Imperial College Healthcare NHS Trust, Fulham Palace Road, London W6 8RF, UK

## Abstract

*Objective*. Diagnostic reliability of prenephroureterectomy ureteroscopy (PNU) for the detection of upper tract carcinoma in situ (CIS) remains unproven in particular and underreported in general. *Methods*. Patients who underwent radical nephroureterectomy (RNU) in a large multicentre retrospective study for upper tract transitional cell carcinoma (UT-TCC) between January 2002 and December 2013 were identified from our hospitals databases. PNU appearances, stage, and grade of ureteroscopic biopsy were compared with final histology results of RNU to assess the diagnostic reliability of PNU for carcinoma in situ (CIS). *Results*. Three hundred patients underwent RNU for UT-TCC. 106 (106/300; 35.3%) of the cohort had PNU using white light with biopsies taken in most (92/106; 86.7%). Postnephroureterectomy histology of the cohort showed CIS in 65 (65/300; 21.6%) patients. Thirty nine of patients with CIS (39/65; 60%) had prenephroureterectomy ureteroscopy biopsies. Out of ten patients with CIS on ureteroscopic biopsies, six did not show CIS on final histopathology (6/10; 60%). Moreover, grading and staging on PNU biopsies of obvious tumours showed a significant nonconcordance with final histopathology of RNU specimen (*P* = 0.02). Overall survival was also shorter in patients with CIS compared with those without; this showed strong statistical significance (*P* = 0.004). *Conclusions*. There is a high incidence of CIS in upper tract with significant underdetection and discordance rate between the histopathology of biopsy samples obtained by white light PNU and resected specimen of radical nephroureterectomy. The presence of concomitant CIS and high stage disease in the upper tract TCC carried a poor prognosis following radical nephroureterectomy.

## 1. Introduction

Upper tract urothelial cell carcinoma (UT-UCC) accounts for approximately 5% of all urothelial cancers with an incidence of 1-2 per 100,000 population/year [1]. The clinical diagnosis of these cancers is made on upper tract imaging (CT urography), urine cytology, and ureteroscopic biopsy. Accurate grading and staging using these modalities form the cornerstone for risk stratifying patients to appropriate treatment options including endoscopic management.

Radical nephroureterectomy (RNU) with excision of cuff of urinary bladder on the tumour bearing side remains the standard-of-care for high grade and high stage disease. There are, however, an increasing number of reports of endoscopic nephron-sparing approaches, particularly in small and low grade tumours [2–5]. Weighing the advantages of endoscopic nephron-sparing approaches against oncological failure is crucial and perhaps the most challenging. The recent EAU guidelines suggest endoscopic nephron-sparing approaches (eNSS) as management options for low grade and low volume disease even in healthy fit patients with normal contralateral kidneys [6]. Grade and stage of tumours are the major determinants of oncological outcomes and this important information on prenephroureterectomy ureteroscopy (PNU) biopsy is critical for selecting patients for safe endoscopic approaches [7].

Presence of concomitant carcinoma in situ (CIS) on PNU could potentially influence decision making as CIS is considered to be a poor prognostic histologic feature in contemporary bladder cancer series [8]. There is a paucity of the reported literature for incidence of carcinoma in situ in upper tract, diagnosis by PNU, and/or suspecting these lesions on abnormal mucosal appearances during ureteroscopy. Nevertheless, its presence in the postresection final nephroureterectomy specimen is associated with increased disease recurrences and progression. Almost one in three patients following RNU was found to have concomitant CIS in a single-institutional study [9] with significant impact on follow-up recurrences. This has been further confirmed by recent reports [10, 11]. McCarron Jr. et al. reported a higher incidence of CIS in close proximity to the obvious tumours [12]. Therefore, the presence of a concomitant CIS should always be actively sought in patients with UT-UUC, in particularly those opting for endoscopic management. There are, however, a number of recognised concerns: limited diagnostic accuracy of white light endoscopy for subtle and early mucosal changes, no protocols for random biopsies, no reliable technique of using photosensitizers for photodynamic diagnosis in upper tract, and discrepancy between PNU biopsy and final radical nephroureterectomy histology and poor yield of PNU biopsy specimen to reliable grade and stage disease. These unresolved issues impede the progress of endoscopic management in UT-UCC.

The aims of the present study are as follows:

1. to report incidence of concomitant CIS in radical nephroureterectomy and assess reliability of white light PNU with biopsy in diagnosing these lesions,
2. to evaluate the impact of concomitant CIS on the oncological outcomes (recurrence-free survival and disease specific and overall survival) of patients with UT-UCC.

## 2. Patients and Methods

### 2.1. Data Source

SUrgical Treatment of Upper tRact urothelial cancErs (SUTURE) group is a large consortium of surgeons with specific interest in the outcomes of UT-TCC. The hospitals of consortium membership cater a relatively stable population of more than 1.5 million of the UK. Histology of each patient is reviewed at local weekly multidisciplinary meetings by uropathologists and the outcome is safely stored in pass word protected computers.

We identified a cohort of patients who underwent RNU for clinically localised UT-UCC between January 2000 and December 2013 using terms “nephroureterectomy” and “ureteroscopy” in cancer multidisciplinary meetings records. The numbers were validated by cross-checking databases maintained independently by the department of pathology of the institutions. Extracted data comprised ten-digit unique community health index number (CHI number for Scotland alone), anonymised date of birth, presenting symptoms, imaging investigations, notes from multidisciplinary meetings, PNU biopsy results, date of surgery, histopathology of the radical surgery, recurrences, and finally the date of death along with underlying and contributory cause of death.

Staging of tumours by TNM system and grade of tumour differentiation according to WHO classification of 1998 [13] were extracted from the histopathology and multidisciplinary records and classified into three tier-numbering categories: (1) G1—low grade, (2) G2—intermediate grade, and (3) G3—high grade [14, 15]. All surgical procedures were performed either by employing laparoscopic or open approaches. Voided urinary cytology specimens were obtained prior to any instrumentations/procedures for the detection of malignant cells. The specimen were immediately sent to pathology laboratory and examined by senior cytologists using conventional methods.

### 2.2. PNU and Ureteroscopic Biopsy Technique

The technique of ureterorenoscopy has been described previously [16]. The consortium members have a special interest in this area including use of photosensitizer in the upper tract [16]. In order to rule out possibility of CIS, normal looking mucosa was not biopsied to rule out the possibility of CIS. Briefly, following standard cystoscopy, white light ureterorenoscopy (semirigid and flexible; 7.5 Fr KARL STORZ Flex-X ureterorenoscope) was carried out to visualize tumors suspected or seen on imaging. A 3-Fr Piranha (Boston Scientific, Natick, MA, USA) cold cup forceps were introduced through the ureteroscope and surface of the lesions was grasped. The grasped tissue is pulled out along with the ureteroscopes. On few occasions stone baskets (Boston Scientific, Natick, MA, USA) or 3 Fr biopsy forceps (KARL STORZ, Germany) or brush forceps were used instead. Multiple biopsies were carried out from all obvious or suspicious areas or both. Random biopsies from normal mucosa were taken from upper urinary tract only if they were reported as suspicious (thickening of ureter, etc.) on CT urogram. The biopsy specimen were fixed in formalin and processed as standard for haematoxylin and eosin staining. The WHO histological grading system was used as described above. All patients in the present study had confirmed diagnosis of upper UT-UC on final histopathology. Radical nephroureterectomy procedures were performed by a standard open approach or transperitoneal laparoscopic route with handling of the lower end of the ureter either endoscopically or by an open method. In most of tumors in the lower end of ureter, open excision was performed except for a selected group where wide excision of cuff of bladder was carried out by a technique described by our group [17].

### 2.3. Follow-Up Protocol

Patients were followed up according to standard protocols at each centre. Follow-up consisted of a history, physical examination, routine blood work, urinary cytology, and flexible cystoscopic inspection of the urinary bladder. Cystoscopy was performed at 3–5 months following nephroureterectomy and annually thereafter. Cross-sectional imaging (abdominal and chest computed tomography or magnetic resonance imaging) was performed at 6 months after surgery and yearly thereafter. Any recurrences were brought back to the multidisciplinary meetings for review of management plans.

### 2.4. Oncological Outcomes

Recurrence was defined as a new urothelial cancer in the bladder/urethra (lower urinary tract recurrence), contralateral upper tract, or local recurrence. Metastasis was recorded separately.

The primary outcome of the study was a diagnostic accuracy of PNU in reliably diagnosing concomitant CIS and the secondary outcome was recurrence-free, cancer specific, and overall survival in men with concomitant CIS in upper tract.

### 2.5. Statistical Analysis

The impact of PNU on the detection of CIS was described as a number of patients and percentages for all categorical variables. The Fisher's exact test and the two-tailed test were used to evaluate the association between PNU (detection of CIS, grade, and stage of UT-UC) and final pathologic parameters. Differences in variables with a continuous distribution across categories were assessed using the Mann-Whitney *U* test. The Kaplan-Meier method was used to calculate survival functions, and differences were assessed with the log rank statistic. Multivariable survival analyses were performed using the Cox proportional hazard regression model. Statistical significance in this study was set as *P* < 0.05. All statistics were completed with SPSS version 21.0 (IBM SPSS Inc., Chicago, IL).

## 3. Results

Three hundred patients underwent radical nephroureterectomy for upper tract transitional cell carcinoma during the study period. The mean age of the cohort was 71.82 ± 8.67 yrs and the majority were male (60.3%). The baseline characteristics of the cohort are shown in Table 1. 106 (106/300; 35.3%) of the cohort had PNU using white light, with biopsies taken in 92/106 (86.7%). None of these patients were suspected to have mucosal changes suggestive of concomitant CIS on PNU.

### 3.1. Prenephroureterectomy Ureteroscopic Diagnosis of CIS

Concomitant carcinoma in situ was found in the final histopathology of nephroureterectomy specimen in 65 (65/300; 21.6%) cases of the cohort (Table 1). Thirty nine (39/65; 60%) of these patients with CIS on final histopathology had white light ureteroscopy. Preureteroscopic voided urinary cytology was negative for malignant cells in 18 (18/29; 62%) of the concomitant CIS cases.

**Figure 1:**
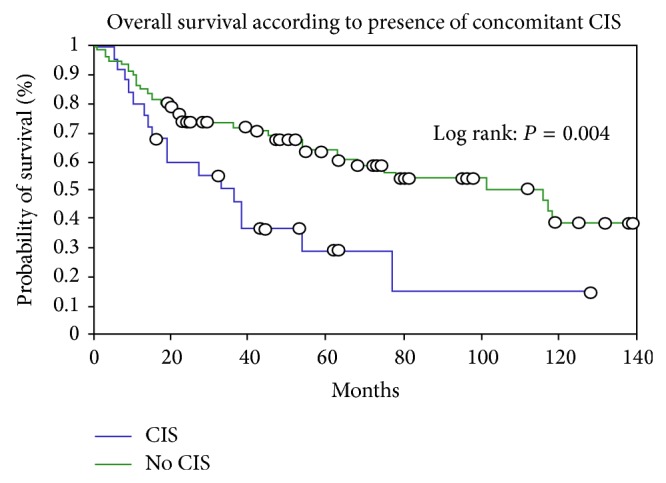
Overall survival according to presence or absence of CIS in the upper urinary tract.

Concomitant CIS was diagnosed on PNU biopsies in 10 (10/39; 15.3%) patients. Out of these, six did not show CIS on final histopathology (6/10; 60%). One of these patients was found to have a single focus of primary CIS on PNU; however, postresection radical nephroureterectomy showed no tumour (false positive). PNU failed to suspect or diagnose CIS in 29 patients (29/39; 84.7%). There were 19 patients in the present study with low grade histology on PNU and with a subsequent diagnosis of CIS on histopathology of nephroureterectomy. These patients could have been suitable for endoscopic management alone based on appearances on white light ureteroscopy and PNU biopsy. Overall survival of those with concomitant CIS was significantly lower than those without (Figure 1; *P* value 0.004).

### 3.2. Diagnostic Accuracy of PNU Compared to Reference Standard for Upper Tract Tumours (Table 2 and Figure 2)

**Figure 2:**
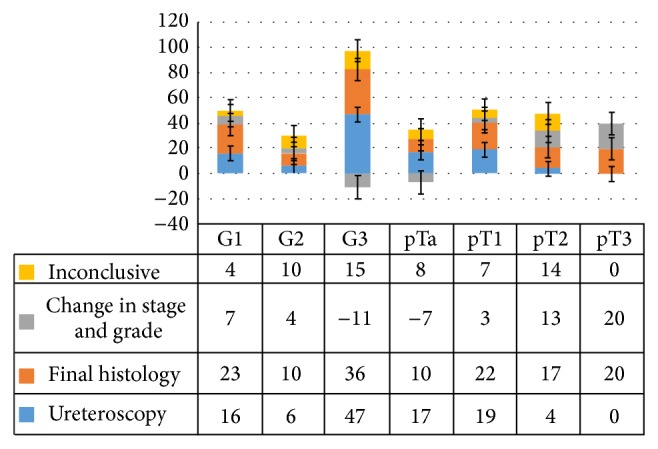
It shows up-/understaging of PNU histology including outcomes of inconclusive PNU biopsy specimens.

Ninety-two patients had PNU biopsies. Only 30 patients (30/92; 32.6%) had concordance between PNU and final nephroureterectomy histology for both stage and grade of disease. Histopathological grading or staging on PNU biopsy samples was difficult and not possible in 29 patients (29/92; 31.5%). Insufficient or poor quality biopsy material remained the main reason. In the remaining cases of the cohort, PNU biopsy grade matched with the final histopathology in 40 (40/92; 43.4%) and was both upgraded and downgraded in 11 (11/92; 11.9%) and 11 (11/92; 11.9%) patients, respectively. Interestingly, being inconclusive or negative for grading on PNU was reported as high grade tumours subsequently on postresection nephroureterectomy histology (Figure 2).

PNU failed to accurately stage UT-UCC in half of the patients. A significant numbers were up staged (36/92; 39.1%) following nephroureterectomy. Staging was not possible for 29 patients (29/92; 31.5%). Moreover, PNU biopsies did not do well in those with higher stage disease (T2 and more; Figure 2).

## 4. Discussion

Several observations in this study are worth noting. A nonconcordance rate of 45% and 62% for grade and stage between PNU and final nephroureterectomy histology, respectively, is much higher than some of the previously reported studies [18, 19]; however, this is similar to others [14, 15]. The concordance rate was not better for higher grade tumours in contrast to the findings of a previous report [20]. In this large multi-institutional study, PNU significantly underdetected concomitant CIS and this remains a major concern. In part, our inability to obtain precise information about CIS may explain natural history of higher recurrences and progression in patients opting for endoscopic management [21–23]. The incidence of concomitant CIS in the present study and previous report [9] underscores the importance of active search for this entity in the upper tract as this may influence the decision making especially making a choice between endoscopic nephron sparing and surgical excision approaches. The rate of CIS varied between 10 and 25% between the centres; however, it is still lower than some of the previous reports. False positive and false negative results of ureteroscopic biopsies have been reported in the previous studies [14, 24–26]. Our own observation from one (false positive) in the present study should be kept in mind while using ureteroscopic biopsy as a tool for selecting patients to endoscopic or extirpative management, in particularly when CIS is the only diagnosis on PNU.

In spite of advanced expertise in fibrooptic upper tract endoscopy, issues with ureteroscopic biopsies such as suboptimal tissue volume, poor quality, lack of standardisation of biopsy technique, and type of preservative for storage of specimen remain unresolved. Wason et al. [27] described a better tissue quality yield of ureteroscopically retrieved biopsy material using BIGopsy forceps in comparison to 3-Fr ureteroscopic forceps. The specimens were better preserved and were of sufficient size (31.2 mm versus 3.5 mm) especially for sessile and flat lesions. Numbers in the study, however, were small and tissue samples obtained from nephroureterectomy specimen were in an* ex vivo* setting. Recently Ritter et al. [28] have reported a lower field of view using BIGopsy forceps again in* ex vivo* settings. Other factors such as irrigation flow and deflectability of endoscopes need to be kept in mind for flexible ureteroscopic and particularly in areas of relatively difficult access. This may explain a large variation which exists in quality of tissue obtained from renal pelvis and ureters as former site is difficult to approach. Whether insertion of ureteral access sheath improves acquisition of better quality tissue as suggested by Gorin et al. [29] needs further study. The quality of tissue retrieved should be a denominator for any technological improvement. The largest samples obtained may not be necessarily of a good quality as they may suffer from lack of architectural preservation due to urothelial denudation [29]. Bultitude et al. [30] suggested use of Bouin's fixative method for preservation of nuclear details of the tissue samples obtained ureteroscopically. The method may be useful for determining the grade of tumour; however, its role in reducing the discordance rate as seen in the present needs further assessment. Obtaining good quality tissue samples from flat and sessile lesions such as CIS poses a difficult challenge in a relatively narrow structure such as ureter. A deeper bite may lead to perforation or higher risk of tumour cell seeding especially in high grade lesions.

Presence of carcinoma in situ is known to be a poor prognostic marker for urinary bladder cancer; however, there are no studies to assess reliability of PNU in diagnosing this entity in the upper tract. Interestingly, if not surprisingly, none of the concomitant upper tract carcinoma in situ lesions as confirmed on histopathology of nephroureterectomy specimen was suspected or diagnosed on PNU. The majority of patients with concomitant CIS had high grade UT-UCC, higher follow-up recurrences, and poor outcome. There were 19 patients in the present study with low grade histology on PNU histology and with a subsequent diagnosis of CIS on histopathology of nephroureterectomy. Potentially, these patients could have been offered endoscopic management and CIS would have been missed. With a low concordance rate for grading and missing of CIS on PNU, proper selection of patients with UT-UCC for endoscopic management becomes a real issue. Additional information from urinary cytology did not improve diagnostic precision in the present study. The role of other urinary biomarkers in conjunction with white light ureteroscopy for the diagnosis of upper tract TCC needs future research. New endoscopic developments such as photodynamic diagnosis [16, 31], narrow band imaging [32], and optical coherence tomography [33] are emerging to address the issue of poor ureteroscopic retrieval of tissues. The diagnostic accuracy of these emerging imaging techniques needs to be assessed using proper gold standard such as final histopathology from nephroureterectomy specimen. These techniques may become part of eNSS approach for upper tract TCC in the future.

We have used a large multi-institutional data with validation of part of the cohort by electronic cross-linkage methodology as described previously [34, 35]. Although all the centres have a strong interest in and experience of endoscopic diagnosis of upper tract urothelial cancers, lack of randomisation to PNU, no PNU biopsies from normal appearing ureters to confirm diagnosis of CIS prior to nephroureterectomy, lack of information on whether CIS was de nova or concomitant, and a small number of patients with CIS on PNU are some of limitations of the present study. The data from present study, however, should encourage reporting and active search to rule out CIS in patients opting for endoscopic management. How best we can achieve this remains a topic of future research and discussions. Furthermore, large tumours obvious on CT urography and those with tight malignant ureteric strictures may not have gone through diagnostic ureteroscopy route and this induces a selection bias in the present study. A further limitation of the findings from the present study could be the possibility that different instruments were used in multi-institutional/multisurgeon nature of the PNU biopsy procedure. Given a high expertise is needed to obtain reliable biopsy tissue in PNU, this may be another source of bias in the present study.

A number of areas of future research in the management of upper tract TCC come under sharp focus in the light of findings of the present study.

1. Prospective protocol driven studies with robust reference standards are urgently needed to assess the diagnostic accuracy of PNU in selecting low grade patients for endoscopic nephron sparing approach.
2. Technological advancements especially role of photodynamic diagnosis and optical coherence tomography need to be assessed in prospective multicentre studies.
3. A consensus on the quality of ureteroscopically retrieved biopsy material including methods of preservation needs to be reached through cross-disciplinary approach.
4. Due to relative rare nature of these tumours, a multicentre international collaborative group with focus on key questions both in research and clinical need to be established.

## 5. Conclusions 

There is a high incidence of CIS in upper tract with significant underdetection and discordance rate between the histopathology of biopsy samples obtained by white light PNU and resected specimen of radical nephroureterectomy, in particularly for the presence of CIS. The presence of concomitant CIS and high stage disease in the upper tract TCC carried a poor prognosis following radical nephroureterectomy.

## Figures and Tables

**Table 1:** Demographic characteristics of the study cohort.

Cohort characteristics		Number (%age)
Total number		300
Number with pre nephroureterectomy ureteroscopy (PNU)		106
Numbers with PNU biopsy		92
Mean age in years (standard deviation)		71.82 ± 8.67
Gender	Male	180 (60%)
	Female	120 (40%)
Stage	pTa	116 (38.7%)
	pT1	83 (27.3%)
	pT2	51 (17%)
	pT3	49 (16.3%)
Grade	G1/G2	89 (29.6%)
	G3	146 (48.6%)
	CIS	65 (21.6%)
Multifocal		143 (47.6%)
Urinary bladder recurrences		83 (27.6%)
Contralateral upper tract recurrence		28 (9.3%)

**Table 2:** Histopathological outcomes of patients with prenephroureterectomy ureteroscopic biopsies.

Characteristics		Prenephroureterectomy biopsy histology numbers (%age)	Nephroureterectomy histology^**^ numbers (%age)
Grade	G1	16 (17.3%)	30 (32.6%)
	G2	6 (6.5%)	20 (21.7%)
	G3	47 (51.08%)	42 (45.6%)
Stage	pTa	17 (18.4%)	11 (11.9%)
	pT1	19 (20.6%)	22 (23.9%)
	pT2	4 (4.3%)	39 (42.3%)
	pT3	0	20 (21.7%)
	Not possible	29 (31.5%)	0
Carcinoma in situ	Concomitant CIS	9 (9.7%)	39 (43.3%)
	Primary CIS	1 (1.08%)	0
Inconclusive	Insufficient material	20 (21.7%)	0
	Other reasons	3 (3.2%)	0

^**^Column contains final results including the inconclusive ureteroscopic biopsies.
